# Mode shifting in school travel mode: examining the prevalence and correlates of active school transport in Ontario, Canada

**DOI:** 10.1186/1471-2458-11-618

**Published:** 2011-08-03

**Authors:** Bonny Yee-Man Wong, Guy Faulkner, Ron Buliung, Hyacinth Irving

**Affiliations:** 1 Faculty of Physical Education & Health, University of Toronto, 55 Harbord Street, Toronto, Ontario, M5S 2W6, Canada; 2 Department of Geography, University of Toronto at Mississauga, 3359 Mississauga Road North, Mississauga, Ontario, L5L 1C6, Canada; 3 Centre for Addiction and Mental Health, 250 College Street, Toronto, Ontario, M5S 2S1, Canada

## Abstract

**Background:**

Studies examining the correlates of school transport commonly fail to make the distinction between morning and afternoon school trips. The purpose of this study was to examine the prevalence and correlates of mode shift from passive in the morning to active in the afternoon among elementary and secondary school students in Ontario, Canada.

**Methods:**

Data were derived from the 2009 cycle of the Ontario Student Drug Use and Health Survey (OSDUHS). 3,633 students in grades 7 through 12 completed self-administered questionnaires. Socio-demographic, behavioural, psychological, and environmental predictors of active school transport (AST) were assessed using logistic regression.

**Results:**

Overall, 47% and 38% of elementary school students reported AST to and from school, respectively. The corresponding figures were 23% and 32% for secondary school students. The prevalence of AST varied temporarily and spatially. There was a higher prevalence of walking/biking found for elementary school students than for secondary school students, and there was an approximate 10% increase in AST in the afternoon. Different correlates of active school transport were also found across elementary and secondary school students. For all ages, students living in urban areas, with a shorter travel time between home and school, and having some input to the decision making process, were more likely to walk to and from school.

**Conclusions:**

Future research examining AST should continue to make the analytic distinction between the morning and afternoon trip, and control for the moderating effect of age and geography in predicting mode choice. In terms of practice, these variations highlight the need for school-specific travel plans rather than 'one size fits all' interventions in promoting active school transport.

## Background

Given the increasing trend of obesity and decline in physical activity in children and youth [1], the journey to and from school presents one source of energy expenditure through the use of non-motorized travel modes such as walking or biking. Children and youth who practice active school transport (typically walking or cycling) have been consistently found to be more physically active overall than children who do not [2]. However, studies consistently report decreasing prevalence of active school transport (AST) [3]. For example, in the Greater Toronto Area, Canada's largest city-region, the prevalence of walking as the usual mode to school decreased from 1986 to 2001 (53% to 42% for children aged 11-13, 39% to 31% for children aged 14-15) [4].

Identifying the prevalence and correlates of active school transport is important for the development of interventions aimed at promoting AST and future surveillance and monitoring of policy effectiveness. Reviews of the literature identify the broad range of correlates of AST that have been studied and highlight that findings are commonly mixed. This is probably due to the heterogeneity of populations and locations being studied [3]. That is, the importance of certain correlates may vary based on geographic location and age group of the population being studied. There have been at least four studies on AST among Canadian children and youth [5-8]. In terms of socio-demographic factors, girls [5,7,8], students with lower socio-economic status [9], and older students [7-9] were consistently found to be less likely to walk or cycle to school.

Only one Canadian study has examined psychological correlates of AST, finding no association between perceived athletic ability, perceived parent encouragement, and perceived weight status and AST [8]. Of growing interest is the concept of childhood independent mobility. Independent mobility is defined as the 'opportunity for children to move around independently without adults as escorts' [10]. Independent mobility is of interest because children with greater independent mobility may spend more time outdoors being physically active [11]. Autonomy with respect to mobility decisions and outcomes has been found to be an important psychological factor impacting on school transport [11-16]. For example, youth who reported their parents knew little or nothing about their whereabouts after school were more likely to actively commute to school [13]. Notably, the impact of whether children and youth are involved in the process of decision-making (which may reflect both independence and motivation for walking) on school transport mode has not been studied.

For the behavioural correlates of active school transport, children and youth who actively travelled to school were consistently found to be more physically active regardless of age, gender, and country [8]. Environmental factors also may play an important role in school transport. Canadian studies have found that youth living in urban areas [7,8] and within a shorter distance to school were more likely to actively commute to school [5,6]. These findings are also consistent with international research [3,17]. Moreover, regional differences in active school transport have been consistently reported [18,19].

More broadly, within the school transport literature internationally, separate analyses for school mode choice in the morning and afternoon has not been a common practice. For example, in the comprehensive review by Sirard and Slater [3], this distinction is not explicitly discussed. In a recent systematic review on the built environment and active school travel, only five out of 14 studies analysed school mode choice in the a.m. and p.m. periods separately [20]. Recent research highlights the importance of making such a distinction [6]. Importantly, no study has examined the correlates of the most common mode shift - shifting from passive modes of travel in the morning to active modes of travel in the afternoon [3,20]. It is important to identify these *mode shifters *since promoting active school transport may be more feasible for these individuals than those being driven both ways. These parents or other caregivers already allow their children to take at least one trip actively. Identifying who these students are may also be helpful in the consideration of interventions that specifically target the a.m. or p.m. period. Apart from some exceptions [21], the prevalence and correlates of both elementary and secondary school students have not commonly been analysed separately. Existing studies [5,7,8] have also relied on convenience samples for their analyses. To address these gaps, the present study uses a provincially representative sample of elementary and secondary school students in Ontario, Canada to examine the following two questions: i) what is the prevalence, and the correlates of school travel mode to school and back home and ii) what are the correlates associated with the school travel choices of students who shift from passive to active modes between the a.m. and p.m. school travel time periods.

## Methods

### Subjects and procedures

Three thousand and six hundred students in grades 7 to 12 in Ontario completed the 2009 cycle of the Ontario Student Drug Use and Health Survey (OSDUHS), a self-administered, anonymous questionnaire, during a class period. The OSDUHS is a repeated cross-sectional survey conducted every two years by the Centre for Addiction and Mental Health, Toronto, Canada, to assess the health behaviours of youth in Ontario. These respondents were selected using a stratified two-stage cluster sampling method that used representative sampling of all grade 7 to 12 students in publicly funded schools in Ontario. Stratified by school type (elementary and secondary school) and by region (*n *= 4; Toronto; Northern Ontario [Parry Sound District, Nipissing District and farther north]; Western Ontario [Hamilton, Peel District, Dufferin County and farther west]; and Eastern Ontario [Ottawa, Leeds-Grenville-Lanark District, Haliburton-Kawartha-Pine Ridge District, Simcoe County, Durham Region, and York Region]), schools were randomly selected first, followed by a random selection of classes of each grade within each selected school. Of the 14,196 students enrolled in selected classes, 9,241 participated in the survey. The student participation rate was 65% (13% absenteeism and 22% unreturned consent forms or parents' refusal). One hundred and twenty-nine respondents were dropped from the dataset, due to failure to report valid responses for age or sex, or because they did not complete at least half of the questionnaire (9,112 included). Of these 9,112 cases, 1,533 elementary students (Grade 7 and 8) and 2,728 secondary (Grade 9 to 12) completed Form B questionnaires that included questions about school transport developed by the authors. One hundred fifty-seven elementary school students did not complete at least half of the school transport section and hence were excluded (1,376 included). Elementary school students had difficulty in providing information on parental education (19.5% missing data), their anthropometric measures (8.8%), and postal code of their residence (6.5%). However, included and excluded subjects were comparable in terms of sex, grade and regions (Cohen's d [22] = 0.12 for sex, 0.15 for grade and 0.09 for regions). Cases in which neither parent's education was available (n = 268 elementary and n = 198 secondary) were recoded to the sample mean of 14.5 years. This construct was developed using an approach similar to that used in previous research [23-25]. After substituting missing data on years of parent's education with the mean value, among 1,376 and 2,728 elementary and secondary students, 1,086 (78.9%) and 2,479 (90.9%) elementary and secondary school students had complete data which was used for addressing the first objective. For the second objective, among 1,086 and 2,479 elementary and secondary school students from the first objective, 741 (61.6%) and 1,960 (77.2%) who did not walk/bike to school in the morning were included. Among students included in the second objective, 102 elementary and 272 secondary school students shifted their travel mode from passive in the morning to active in the afternoon. Ethical approval for the study was granted by the institutional research ethics committees at CAMH, York University, as well as at eleven Ontario district school boards.

### Instruments

#### School transport

Travel mode to school in the morning and from school in the afternoon was assessed separately. Respondents were asked 'how do you usually travel i) to and ii) from school?' School transport modes for the morning and afternoon were reclassified into a categorical variable with two categories: walking/biking and other. This mirrors the practice in much of the literature to examine travel behaviour across a broad classification of active and passive travel modes [3]. Mode shifters were defined as those not walking to school in the morning but walking back home in the afternoon.

#### Correlates

Socio-demographic (eight variables), behavioural (six variables), psychological (four variables), and environmental (seven variables) correlates were assessed (n = 25 in total).

#### Socio-demographic

Demographic variables such as sex and grade (Grade 7 to 12) were examined. BMI was computed based on self-reported height and weight. The weight status (normal, overweight and obese) was defined according to age- and sex-specific international BMI (IOTF) cut-offs [26]. Years of highest parental education, a socio-economic status indicator, was assessed considering paternal and maternal education. Parents' place of birth and having a sibling(s) were also assessed. With respect to the transportation context, driver licensing and number of household cars were examined.

#### Behavioural

Students were asked 'On how many of the last seven days were you physically active for a total of at least 60 minutes each day?' Examples such as brisk walking, soccer, swimming were given. The Centers for Disease Control and Prevention (CDC) physical activity recommendation for adolescents [27] is at least 60 minutes of MVPA on most days of the week. In the context of active school transport, physical activity levels over five school days may be more relevant. Therefore, Moderate to Vigorous Physical Activity (MVPA) was classified as zero to four days, and five days or more. Self-reported screen-time was based on the current recommendation of 2 hours or less of screen time daily [28] and grouped as 2 hours per day or less, and more than 2 hours per day.

To examine whether there were other behavioural correlates of active school transport, smoking in the past 12 months was asked and categorised as non-smokers (never in lifetime)/smoked but not in past 12 months) and smokers (a few puffs to a whole cigarette everyday). Drinking in the past 12 months was categorised as non-drinkers (never/drank but not in past 12 months) and drinkers (just had a sip of alcohol to see what it's like to almost everyday).

Working part-time and participation in extracurricular activities may incur a time cost and reduce the likelihood of active school transport. Moreover, students and their households may have other destinations than home in the morning or afternoon that may affect school travel mode choice. Extracurricular participation was categorised as yes and no. For secondary students, working part-time was grouped as 'don't work for pay outside home,' 5 hours/week or less, and 6 hours/week or more.

#### Psychological

To measure perceived weight status, respondents were asked 'do you think of yourself as being too thin, about the right weight, or too fat?'. Perceived physical health was measured by asking 'how would you rate your physical health?' with options of excellent, very good, good, fair, and poor and re-grouped into excellent/very good/good and fair/poor.

The extent of parental supervision was asked with the question, 'In your free time away from home, how often does at least one of your parents know where you are?'. Responses were regrouped as never/rarely/sometimes and often/always. The question of autonomy with respect to school travel was addressed by asking, 'How often do you get to decide how you travel to school and back home'. Responses were reclassified as never/rarely/sometimes and often/always.

#### Environmental

Travel time to school and from school were measured separately and categorised as 0-15 minutes, 16-30 minutes and 31 minutes or more. Longer travel time implies longer distance, greater cost (in terms of temporal value), and more effort required for each mode. The same travel time implies greater (physical) effort required for active compared to passive modes (e.g., more physical effort required for walking for an hour than driving/being driven for an hour) and longer travel distance for passive than active modes. Given that parental decision on their child's school travel mode mainly is based on convenience [29], travel time may be an important correlate of school travel. The likelihood of an active school travel mode is hypothesized to decrease with travel time whereas the likelihood of a passive mode is hypothesized to increase with travel time.

Moving home or changing school in the past five years was modeled categorically; residential mobility may act as a latent indicator of neighbourhood familiarity (i.e., familiarity produces active travel). Other environmental characteristics included living in an urban or rural area (based on postal codes) and school affiliation (catholic or no religion) which has been suggested as a correlate of AST [15]. Differences in religious affiliation may be a broader indicator of a difference in school transport policy between different school boards. Regional variation in AST was also examined.

### Data analysis

All analyses used Taylor series methods to account for the complex survey design. Logistic regression was used to identify the correlates of active school transport separately for the morning and afternoon and for students who mode shifted from passive in the morning to active in the afternoon. Two models were created: i) unadjusted models where the effects of each variable on active school transport or mode shift were examined separately and ii) fully adjusted models where only significant correlates in the unadjusted models were included. Physical activity and weight status as well as demographic (grade and sex) variables were included in all models. All analyses were stratified by elementary and secondary school students due to potential differences in independence, availability and use of travel modes, and correlates [17]. All analyses were conducted using Stata 11.

## Results

Tables 1 and 2 summarize the descriptive information for the sample and the prevalence of active school transport. The overall prevalence of active school transport ranged from 38% in the morning to 47% in the afternoon among elementary school students. The corresponding figures were 23% and 32% for secondary school students. The lowest prevalence of walking/biking to and from school in elementary school students were 23% in Northern Ontario and 30% in Eastern Ontario, respectively, whereas the greatest proportions were 60% and 68% in Toronto, correspondingly. The prevalence of walking/biking to and from school in secondary school students ranged from 20% and 24% in the North to 30% and 39% in Toronto, respectively. Among secondary students in the sample, 42.2% held a drivers license, considerable potential exists for secondary students to drive alone or with others either to or from school.

**Table 1:** Descriptive statistics for elementary and secondary school students

	Elementary n (%)	Secondary n (%)	p-value
**Socio-demographic**			
Grade			
Grade 7	506 (48.2)		-
Grade 8	583 (51.8)		
Grade 9		590 (21.6)	
Grade 10		654 (23.4)	
Grade 11		597 (24.3)	
Grade 12		638 (30.8)	
Sex (female)	604 (48.2)	1272 (47.9)	0.91
Weight status (overweight/obese)	241 (26.7)	620 (24.8)	0.42
Years of highest parent education (yrs) (mean(SD))	14.6 (1.6)	14.5 (1.7)	0.17
Parent born abroad (yes)	229 (32.2)	719 (33.0)	0.83
Having sibling (yes)	1011 (92.8)	2328 (93.9)	0.44
Having car at home (yes)	1053 (95.6)	2424 (97.0)	0.13
Having driver license (yes)	-	1046 (42.2)	-
**Behavioural**			
MVPA (≥5 days/wk)	568 (53.9)	1105 (42.6)	< 0.001
Screen time (> 2 hours/day)	539 (49.6)	1518 (61.2)	< 0.001
Smoking in the past 12 months (occasional/regular smokers)	49 (5.3)	572 (22.4)	< 0.001
Drinking in the past 12 months (occasional/regular drinkers)	567 (52.1)	1962 (78.8)	< 0.001
Part-time job			
Did not work outside home	-	1302 (53.5)	-
1-5 hours/wk	-	301 (11.2)	
≥6 hours/wk	-	876 (35.3)	
Extracurricular participation (yes)	838 (77.6)	1755 (70.8)	0.06
**Psychological**			
Perceived physical health (excellent/good)	74 (7.6)	400 (17.7)	< 0.001
Perceived weight status			
About right	779 (73.9)	1699 (65.9)	< 0.001
Too thin	84 (6.3)	218 (10.2)	
Too fat	216 (19.8)	555 (23.9)	
Decision-making on mode choice (often/always)	340 (3.1.5)	956 (40.4)	0.001
Parents don't know where their children are in free time away from home (sometimes/seldom/never)	74 (7.9)	348 (15.9)	< 0.001
**Environmental**			
Travelled to school			
0-15 minutes	708 (68.3)	1570 (63.5)	0.13
16-30 minutes	210 (18.7)	552 (23.9)	
≥31 minutes	168 (13.0)	357 (12.6)	
Travelled from school			
0-15 minutes	646 (61.4)	1202 (48.4)	< 0.001
16-30 minutes	253 (22.7)	738 (30.6)	
≥31 minutes	184 (16.0)	533 (20.9)	
Living in urban area (urban)	857 (84.7.0)	2075 (83.7)	0.86
Regions			
Toronto	112 (21.6)	256 (16.6)	0.07
North	57 (5.7)	199 (6.6)	
West	304 (43.9)	857 (43.6)	
East	613 (28.9)	1167 (33.2)	
Catholic school (yes)	376 (38.9)	935 (36.4)	0.80
Changing school in the last five years (yes)	419 (43.5)	752 (30.2)	< 0.001
Moving home in the last five years (yes)	513 (48.5)	1031 (44.5)	0.24

**Table 2:** Prevalence of active school transport in Ontario

	Secondary				Elementary			
	To school		From school		To school		From school	
	Walk/bike	Other	Walk/bike	Other	Walk/bike	Other	Walk/bike	Other
Toronto	69 (29.8)	187 (70.2)	91 (38.9)	165 (61.1)	65 (59.6)	47 (40.4)	75 (68.4)	37 (31.6)
North	46 (20.0)	153 (80.0)	53 (24.0)	146 (76.0)	11 (22.6)	46 (77.4)	18 (34.4)	39 (65.6)
West	170 (20.9)	687 (79.1)	265 (30.5)	592 (69.5)	127 (39.9)	177 (60.1)	161 (49.9)	143 (50.1)
East	234 (22.5)	933 (77.5)	350 (31.5)	817 (68.5)	142 (23.6)	471 (76.4)	178 (29.7)	435 (70.3)
Total	519 (22.9)	1960 (77.2)	759 (31.8)	1720 (68.2)	345 (38.4)	741 (61.6)	432 (47.2)	654 (52.8)

	*p *= 0.18		*p *= 0.27			*p *== 0.03		*p *= 0.04

Among elementary school students, more years of parental education (OR_to_= 0.83; 95% CI: 0.75-0.92 and OR_fr_= 0.86; 95% CI: 0.78-0.96), longer travel time (OR_to16-30 m_= 0.40; 95% CI: 0.20-0.80, OR_to≥31 m_= 0.11; 95% CI: 0.03-0.32, OR_fr16-30 m_= 0.45; 95% CI: 0.25-0.81, and OR_fr≥31 m_= 0.40, 95% CI: 0.19-0.85), and living in the North (OR_to_= 0.27; 95% CI: 0.10-0.70 and OR_fr_= 0.31; 95% CI: 0.10-0.98) were negatively correlated with walking/biking either to or from school whereas living in urban areas was positively correlated with walking/biking either to or from school (OR_to_= 3.00; 95% CI: 1.29-6.98 and OR_fr_= 4.84; 95% CI: 2.05-11.42) (Table 3). Physical activity (OR_to_= 1.27; 95% CI: 0.95-1.71 and OR_fr_= 0.90; 95% CI: 0.59-1.36) and weight status (OR_to_= 0.83; 95% CI: 0.60-1.33 and OR_fr_= 0.90; 95% CI: 0.59-1.36) were not associated with walking/biking to or from school. Better perceived health and being involved in the school travel decision making process were only correlated positively with active school transport in the afternoon (OR_ph_= 2.20; 95% CI: 1.23-3.92 and OR_decide_= 1.73; 95% CI: 1.11-2.69, respectively). In contrast, the presence of a household car was negatively correlated with active school transport in the morning (OR_to_= 0.22; 95% CI: 0.08-0. 61).

**Table 3:** Individual, behavioural, psycho-social, and environmental correlates of active school transports in elementary school students

	To school				From school			
	Unadjusted OR	95% CI	Adjusted OR	95% CI	Unadjusted OR	95% CI	Adjusted OR	95% CI
**Socio- demographic**								
Grade								
Grade 7	1		1		1		1	
Grade 8	0.96	0.71-1.29	0.83	0.59-1.16	1.01	0.74-1.38	0.95	0.65-1.38
Sex								
Boys	1		1		1		1	
Girls	1.04	0.77-1.40	1.00	0.72-1.37	1.01	0.77-1.33	0.85	0.59-1.23
Weight status								
Normal weight	1		1		1		1	
Overweight/Obese	1.05	0.76-1.46	0.83	0.60-1.33	1.21	0.88-1.66	0.90	0.59-1.36
Years of highest parent education	0.87**	0.78-0.97	0.83**	0.75-0.92	0.90*	0.82-0.99	0.86**	0.78-0.96
Parent born abroad								
Both/one parent born in Canada	-	-			1		1	
Both born abroad	-	-			1.77*	1.06-2.97	0.97	0.53-1.79
Having car at home								
No	1		1		1		1	
Yes	0.20***	0.11-0.37	0.22**	0.08-0.61	0.25***	0.12-0.50	0.34	0.10-1.09
**Behavioural**								
MVPA								
0-4 day/wk	1		1		1			
≥5 days/wk	0.91	0.70-1.19	1.27	0.95-1.71	0.85	0.60-1.19	1.19	0.77-1.84
**Psychological**								
Perceived physical health								
Fair/poor	-	-			1		1	
Excellent/very good/good	-	-			1.90*	1.11-3.24	2.20*	1.23-3.92
Decision-making on mode choice								
Never/rarely/ sometimes	1		1		1		1	
Often/always	1.70**	1.18-2.46	1.35	0.89-2.05	2.04***	1.43-2.90	1.73**	1.11-2.69
**Environmental**								
Travelled to/from school								
0-15 minutes	1		1		1		1	
16-30 minutes	0.38***	0.20-0.70	0.40**	0.20-0.80	0.38**	0.21-0.67	0.45*	0.25-0.81
≥31 minutes	0.07***	0.03-0.20	0.11**	0.03-0.32	0.23***	0.11-0.47	0.40*	0.19-0.85
Living in urban area								
Rural	1		1		1		1	
Urban	5.17***	2.14-12.48	3.00**	1.29-6.98	5.72***	2.60-12.59	4.84***	2.05-11.42
Regions								
Toronto	1		1		1		1	
North	0.25*	0.08-0.77	0.27*	0.10-0.70	0.27*	0.09-0.86	0.31*	0.10-0.98
West	0.50	0.24-1.04	0.71	0.31-1. 60	0.43*	0.19-0.97	0.71	0.27-1.85
East	0.24**	0.10-0.57	0.43	0.15-1.22	0.20**	0.08-0.51	0.40	0.12-1.28

*** *p *< 0.001;** *p *< 0.01;* *p *< 0.05

OR: odds ratio; CI: confidence interval. Only the unadjusted and adjusted ORs and CIs of variables are included in the final models reported in this table.

Table 4 shows the correlates of active school transport for secondary school students. Female students (OR_to _= 0.66; 95% CI: 0.49-0.89 and OR_fr _= 0.74; 95% CI: 0.55-0.99) and those working part-time (OR_to≥6 hr/wk _= 0.65; 95% CI: 0.45-0.93 and OR_fr≥6 hr/wk _= 0.62; 95% CI: 0.44-0.86) were less likely to actively commute to school in either the morning or afternoon. Living in an urban area (OR = 2.67; 95%CI: 1.22-5.82) and contributing to the school travel decision was positively correlated with walking/biking from school (OR = 1.52; 95%CI: 1.13-2.04). Adolescents who walked/biked to school were more physically active (OR = 1.39; 95%CI: 1.01-1.90), had shorter travel times (OR_16-30 min _= 0.84; 95%CI: 0.59-1.19 and OR_≥31 min _= 0.23; 95%CI: 0.13-0.38), and had moved house in the past five years (OR = 1.33; 95%CI: 1.05-1.70). Self-reported weight status was not associated with either morning or afternoon school trips (OR_to_= 0.99; 95% CI: 0.74-1.33 and OR_fr_= 0.94; 95% CI: 0.70-1.27).

**Table 4:** Individual, behavioural, psycho-social, and environmental correlates of active school transports in secondary school students

	To school				From school			
	Unadjusted OR	95% CI	Adjusted OR	95% CI	Unadjusted OR	95% CI	Adjusted OR	95% CI
**Socio- demographic**								
Grade								
Grade 9	1		1		1		1	
Grade 10	0.96	0.69-1.35	0.95	0.67-1.35	0.97	0.72-1.31	1.03	0.73-1.44
Grade 11	1.08	0.76-1.53	1.14	0.77-1.69	1.15	0.80-1.65	1.26	0.81-1.97
Grade 12	0.70	0.49-1.00	0.71	0.48-1.05	0.73	0.53-1.01	0.83	0.56-1.24
Sex								
Boys	1		1		1		1	
Girls	0.70*	0.52-0.94	0.66**	0.49-0.89	0.79	0.60-1.03	0.74*	0.55-0.99
Weight status								
Normal weight	1		1		1		1	
Overweight/ Obese	1.02	0.76-1.36	0.99	0.74-1.33	0.94	0.73-1.21	0.94	0.70-1.27
**Behavioural**								
MVPA								
0-4 day/wk	1		1		1		1	
≥5 days/wk	1.41*	1.04-1.90	1.39*	1.01-1.90	1.27	0.98-1.66	1.21	0.90-1.62
Smoking in the past 12 months								
Did not smoke					1		1	
Occasional/regular smoker					0.74*	0.56-0.98	0.76	0.56-1.03
Part-time job								
Did not work outside home	1		1		1		1	
1-5 hours/wk	0.72	0.48-1.07	0.76	0.52-1.11	0.67**	0.47-0.97	0.80	0.57-1.11
≥6 hours/wk	0.58**	0.41-0.81	0.65*	0.45-0.93	0.55***	0.41-0.75	0.62**	0.44-0.86
**Psychological**								
Decision-making on mode choice								
Never/rarely/ sometimes	-				1		1	
Often/always	-	-			1.57**	1.21-2.05	1.52**	1.13-2.04
Parents don't know where their children are in free time away from home								
Always/usually	1		1					
Sometimes/seldom/never	1.43*	1.00-2.04	1.36	0.94-1.97				
**Environmental**								
Travelled to/from school								
0-15 minutes	1		1		1		1	
16-30 minutes	0.91	0.63-1.33	0.84	0.59-1.19	0.92	0.66-1.28	0.95	0.67-1.34
≥31 minutes	0.24***	0.14-0.40	0.23***	0.13-0.38	0.60**	0.42-0.85	0.72	0.51-1.03
Living in urban area								
Rural	-				1		1	
Urban	-	-			3.17**	1.48-6.78	2.67*	1.22-5.82
Regions								
Toronto	1		1		1		1	
North	0.54	0.28-1.05	0.70	0.37-1.30	0.46*	0.22-0.94	0.58	0.28-1.25
West	0.60*	0.37-0.96	0.69	0.42-1.13	0.62	0.36-1.08	0.93	0.52-1.62
East	0.64	0.40-1.03	0.78	0.47-1.28	0.67	0.40-1.13	0.93	0.53-1.63
Catholic school								
No	1		1		-			
Yes	0.69*	0.48-0.98	0.75	0.53-1.04	-	-		
Changing school in the last five years								
No	1		1		1		1	
Yes	1.39*	1.03-1.84	1.19	0.87-1.63	1.44**	1.10-1.88	1.35	0.99-1.82
Moving home in the last five years								
No	1		1		1		1	
Yes	1.37**	1.10-1.72	1.33*	1.05-1.70	1.27*	1.02-1.58	1.11	0.86-1.43

*** *p *< 0.001;** *p *< 0.01;* *p *< 0.05

OR: odds ratio; CI: confidence interval. Only the unadjusted and adjusted ORs and CIs of variables are included in the final models reported in this table.

In terms of mode shift, both elementary and secondary school students who lived in urban areas (OR_ele_= 3.58; 95%CI: 1.00-12.90 and OR_sec_= 3.24; 95%CI: 1.18-8.85) and decided on their school travel mode (OR_ele_= 2.43; 95%CI: 1.23-4.80 and OR_sec_= 2.42; 95%CI: 1.61-3.63) were more likely to shift their travel mode from passive in the morning to active in the afternoon (Table 5). Among secondary school students, students with a shorter travel time to school were more likely to shift their mode from passive in the morning to active in the afternoon (OR_16-30 m _= 0.11; 95% CI: 0.03-0.40 and OR_≥31 m _= 0.07; 95% CI: 0.02-0.29). Only elementary school students with better perceived health were more likely to shift their travel mode to active (OR = 2.97; 95%CI: 1.00-8.81). Elementary school students whose parents did not know where they were in their free time were more likely to shift their travel mode from passive to active (OR = 1.87; 95%CI: 1.00-3.49). For both elementary and secondary school students, there was no difference in the likelihood of mode shift between students who were physically active for five days per week or more and those who were active less than five days per week (OR_ele _= 1.11 and 95% CI: 0.62-2.00 and OR_sec _ = 0.74; 95% CI: 0.45-1.21).

**Table 5:** Individual, behavioural, psycho-social, and environmental correlates of mode shift

	Elementary				Secondary			
	Unadjusted OR	95% CI	Adjusted OR	95% CI	Unadjusted OR	95% CI	Adjusted OR	95% CI
**Socio- demographic**								
Grade								
Grade 7	1		1		-	-	-	-
Grade 8	0.85	0.58-1.25	0.83	0.51-1.37	-	-	-	-
Grade 9	-	-	-	-	1		1	
Grade 10	-	-	-	-	0.87	0.55-1.38	0.69	0.40-1.16
Grade 11	-	-	-	-	1.11	0.68-1.81	0.93	0.50-1.73
Grade 12	-	-	-	-	0.81	0.48-1.38	0.80	0.42-1. 52
Sex								
Boys	1		1		1		1	
Girls	0.98	0.63-1.54	0.83	0.44-1.58	0.96	0.67-1.37	0.70*	0.49-1.00
Weight status								
Normal weight	1		1		1		1	
Overweight/Obese	1.67	0.97-2.87	1.11	0.62-2.00	0.91	0.62-1.32	0.74	0.45-1.21
Parent born abroad								
Both/one parent born in Canada	1		1					
Both born abroad	1.76*	1.05-2.98	1.35	0.71-2.55				
**Behavioural**								
MVPA								
0-4 day/wk	1		1		1		1	
≥5 days/wk	0.79	0.43-1.44	0.99	0.44-2.20	1.05	0.72-1.55	0.84	0.55-1.28
Part-time job								
Did not work outside home	-				1		1	
1-5 hours/wk	-	-			0.73	0.42-1.29	1.06	0.58-1.92
≥6 hours/wk	-	-			0.68*	0.47-0.99	0.71	0.45-1.12
**Psychological**								
Perceived physical health								
Fair/poor	1		1		-			
Excellent/very good/good	2.86**	1.42-5.76	2.97*	1.00-8.81	-	-		
Decision-making on mode choice								
Never/rarely/sometimes	1		1		1		1	
Often/always	3.00***	1.81-4.96	2.43***	1.23-4.80	2.29***	1.64-3.20	2.42***	1.61-3.63
Parents don't know where their children are in free time away from home								
Always/usually	1		1					
Sometimes/seldom/never	2.64**	1.46-4.77	1.87*	1.00-3.49				
**Environmental**								
Travelled to school								
0-15 minutes	1		1		1		1	
16-30 minutes	0.32*	0.11-0.94	0.11***	0.03-0.40	0.38**	0.21-0.67	0.16***	0.09-0.30
≥31 minutes	0.32*	0.11-0.95	0.07***	0.02-0.29	0.10***	0.03-0.27	0.03***	0.01-0.10
Travelled from school								
0-15 minutes	-				1		1	
16-30 minutes	-	-			1.79**	1.23-2.60	4.32***	2.82-6.60
≥31 minutes	-	-			1.87**	1.23-2.85	10.49***	6.44-17.09
Living in urban area								
Rural	1		1		1		1	
Urban	3.79*	1.14-12.60	3.58*	1.00-12.90	5.60**	1.97-15.89	3.24*	1.18-8.85
Regions								
Toronto	1		1		-			
North	0.52	0.17-1.59	0.65	0.19-2.21	-	-		
West	0.66	0.27-1.60	0.85	0.29-2.48	-	-		
East	0.26**	0.10-0.66	0.42	0.15-1.19	-	-		
Changing school in the last five years								
No	-				1		1	
Yes	-	-			1.48*	1.04-2.10	1.43	0.96-2.10

*** *p *< 0.001;** *p *< 0.01;* *p *< 0.05

OR: odds ratio; CI: confidence interval. The unadjusted and adjusted ORs and CIs of variables only included in the final models are reported in this table.

## Discussion

This study examined i) the prevalence and correlates of active transport to and from school separately and ii) the correlates of mode shift from passive in the morning to active in the afternoon. In terms of the first purpose, the overall prevalence of walking/biking to and from school was 23% and 32%, respectively, for secondary school students and correspondingly 38% and 47% for elementary school students which is higher than that typically reported in the USA [12,18,19,30,31], similar to Australia [32] and New Zealand [33] but lower than European countries [34-36]. The overall prevalence is also consistent with Canadian literature [4,7,8]. The prevalence of active school transport also varied across regions. In the present study, the highest prevalence was found in Toronto. This is perhaps not unexpected given the proliferation of neighbourhood schools within neighbourhoods established throughout the 19^th ^century, the pre-automobile era. Such neighbourhoods would be characterised by a gridded street network and diverse and highly dense land use that facilitates non-motorised travel modes.

There was a higher prevalence of walking/biking either to or from school found for elementary school students than for secondary school students. Elementary schools may be located closer to students' homes, in our data, elementary school students had significantly shorter travel times than secondary school students (analysis not shown). Different correlates of active school transport were also found across elementary and secondary school students. In secondary school students, not working a part-time job, being male, and being physically active were positively correlated with active school transport whereas higher parental education, having a car(s), and poorer perceived health were negatively associated with active school transport in elementary school students.

To the best of our knowledge, the addition of decision making as a correlate has not been assessed before. This is an important correlate of active school transport in this sample. This supports the hypothesis of Panter et al. [17] that parents, the key decision makers, may be influenced by children's opinions when making a travel mode choice. Being involved in the decision making process on school travel mode may reflect both independent mobility and a positive attitude towards walking/biking to school which have been suggested to increase the likelihood of active school transport [14,15,37,38]. These data provide an interesting direction for interventions in actively engaging students as advocates for active modes of school transport. Child and youth involvement in school travel decision making may also be influenced by parental concern about safety issues and their trust in the spatial and other capabilities of children and youth as they navigate through neighbourhoods. Future studies should identify the factors (e.g., personal factors such as motivation for walking and attitudinal factors, such as parental attitudes towards walking and whether their neighbourhood is suitable for walking [e.g., their preference on place of residence]), that may facilitate child and youth involvement in school travel decision-making.

### Shift from motorized modes in the morning to active in the afternoon

This is the first study specifically examining the correlates of mode shift from motorized alternatives in the morning to active in the afternoon. Approximately 10% of both elementary and secondary school students were mode shifters. This represents a sizeable proportion of students that may be an interesting target for understanding how to intervene on travel practices to encourage a larger share of students to take active modes for at least one part of the daily school trip. Living in an urban area, having an input into the decision making process, and having a shorter travel time to school were associated with active school travel in the morning and in the afternoon. Additionally, these variables were also associated with mode shift for both elementary and secondary school students.

The mode shift may be partially explained by parent's schedules and resource accessibility. Elementary school students with mothers who commuted to work in the morning were less likely to walk/bike to school, suggesting that a parent's temporal constraints and desire for convenience affect school travel mode choice [39]. In the afternoon, working parents may not be available to pick children up after school [40], leaving these children and youth with a requirement to find an alternative way to get home. Walking may be feasible for short trips along enabling infrastructures (well-connected street system with signalized intersections), and when they are permitted and prefer to do so. For example, in our sample, more students living in urban areas shifted their travel mode than in rural areas (17.6% vs. 5.9% and 17.3% vs. 1.8% for elementary and secondary school students, correspondingly, p-value < 0.001). Similarly, 20% of elementary and secondary school students being driven to school in 0-15 minutes walked back home whereas about 5% of students who were driven to school in 15 minutes or more shifted their mode in the afternoon (p-value < 0.001). Future studies should further examine the psychosocial (e.g., attitudes towards active commuting to school); household (e.g., parents' scheduling and travel mode to work); and environmental characteristics associated with the mode shifting behaviour reported here.

The present study shows the importance of examining mode shift as this is a group of students demonstrating different travel behaviours at different times of the school day. Focusing on mode shift raises the consideration as to whether interventions might be more effective by targeting either the a.m. or p.m. periods. Mode shifters were more likely to live within walking distance from school. Promoting active school transport in the morning may be more feasible in these mode shifters than those being driven both ways because, for example, parents or other caregivers are already accustomed to allowing their children to take an unescorted trip home in the afternoon.

### Limitations

The nature of cross-sectional study makes causal inference impossible. Our findings solely relied on self-report. Accelerometer may measure active school transport and physical activity more accurately. Substantial missing data on selected variables was found in both elementary and secondary school students; however, included and excluded subjects were comparable in terms of sex, grade and region. The strengths of the present study include a representative sample of Canadian students at a provincial level and analysing morning and afternoon school trips separately demonstrating the variation across time particularly in secondary school students.

Given that distance is the strongest and consistent correlate of active school transport [20], the inclusion of this variable in the analyses would have been informative. However, in this dataset, self-reported distance was not reported nor did we have access to the home and school location of respondents. Time was used as an indicator of the generalized cost of a particular travel mode but the inclusion of time in the specified models would have been strengthened if we had information on the travel times for each alternative transport mode.

### Implications

The spatial, temporal and age-related variations in AST highlight the need for school-specific travel plans rather than 'one size fits all' interventions in promoting active school transport. Engaging children and youth in school transport decision-making may be an important and under-examined process for influencing school transport as well as overall physical activity. A significant proportion of children and youth can be described as mode shifters in moving to active modes of transport in the afternoon, and this natural shift might be an opportunity for the targeting of future interventions; particularly interventions seeking to maximise opportunities for youth physical activity in the after-school period [41]. School transport considerations will be integral to the success of such interventions.

## Conclusions

In summary, our findings demonstrate that the prevalence of active school transport varies across time and region. Similarly, the correlates of school transport vary across time and age groups. Future research examining school transport should continue to make the analytic distinction between the morning and afternoon trip, and control for the moderating effect of age and geography in predicting mode choice.

## List Of Abbreviations

AST: Active school transport; CDC: Centers for Disease Control and Prevention; OSDUHS:Ontario Student Drug Use and Health Survey

## Competing interests

The authors declare that they have no competing interests.

## Authors' contributions

BYW analyzed and interpreted the data and developed the first draft of the manuscript. GF and RB contributed to the conception and the design of the study. HI contributed to the analysis of the data. All authors provided critical feedback during manuscript development. Each author has read and approved the final manuscript.

## Authors' information

BYW is a doctoral student in the Faculty of Physical Education and Health, University of Toronto and is a *CIHR/HSFC Fellow in Population Intervention for Chronic Disease Prevention*. GF is an Associate Professor in Faculty of Physical Education and Health, University of Toronto. RB is an Assistant Professor in the Department of Geography, University of Toronto. HI was a research analyst with the Centre for Addiction and Mental Health (CAMH) at the time of this research.

## Pre-publication history

The pre-publication history for this paper can be accessed here:

http://www.biomedcentral.com/1471-2458/11/618/prepub
